# The EcoData Retriever: Improving Access to Existing Ecological Data

**DOI:** 10.1371/journal.pone.0065848

**Published:** 2013-06-13

**Authors:** Benjamin D. Morris, Ethan P. White

**Affiliations:** Department of Biology and The Ecology Center, Utah State University, Logan, Utah, United States of America; Michigan State University, United States of America

## Abstract

Ecological research relies increasingly on the use of previously collected data. Use of existing datasets allows questions to be addressed more quickly, more generally, and at larger scales than would otherwise be possible. As a result of large-scale data collection efforts, and an increasing emphasis on data publication by journals and funding agencies, a large and ever-increasing amount of ecological data is now publicly available via the internet. Most ecological datasets do not adhere to any agreed-upon standards in format, data structure or method of access. Some may be broken up across multiple files, stored in compressed archives, and violate basic principles of data structure. As a result acquiring and utilizing available datasets can be a time consuming and error prone process. The EcoData Retriever is an extensible software framework which automates the tasks of discovering, downloading, and reformatting ecological data files for storage in a local data file or relational database. The automation of these tasks saves significant time for researchers and substantially reduces the likelihood of errors resulting from manual data manipulation and unfamiliarity with the complexities of individual datasets.

## Introduction

Research in many areas of ecology increasingly relies on the use of data that has already been collected. The use of available data can save time and money by avoiding the re-collection of data, allow questions that would otherwise be intractable to be addressed, and enable prompt recommendations to policy makers in situations where rapid decisions are necessary. While not all areas of ecology are well suited to the use of existing data, those that are have become increasingly limited by the quality and quantity of relevant data that can be acquired [1].

In recent years, ecologists have seen an enormous increase in the amount of data that is publicly available, including: 1) broad scale coordinated data collection efforts such as the North American Breeding Bird Survey [2], the Forest Inventory and Analysis Program [3], and the new National Ecological Observatory Network (http://neoninc.org), which are designed to conduct widespread monitoring of continental-scale processes relevant to biodiversity, climate change, and other ecological concerns; 2) compilations of data from the literature that allow the results of research on individual species or locations to be used more broadly for meta-analysis, such as databases of body size [4,5], life history [6,7], and community composition [8]; 3) complete datasets from individual local scale field projects such as the Portal Project [9] and data on vegetation plots at Mount St. Helens [10]; and 4) data associated with individual publications, provided in supplementary material and through repositories like Dryad (http://www.datadryad.org) resulting from increasing journal requirements for data deposition [11]. As a result, ecological research is becoming increasingly limited not by the availability of data, but by the rate at which that data can be accessed, organized, and analyzed.

Ecologists are still in the process of addressing the challenges associated with this sudden deluge of open data [12,13]. One of the major challenges is that most ecological datasets do not adhere to any agreed-upon standards in format, data structure or method of access [12]. This is despite concerted efforts to improve the structure and usability of ecological data [12,13]. To assist ecologists in quickly and easily accessing and utilizing available data, we have developed the EcoData Retriever (http://ecodataretriever.org), a software package that automatically downloads ecological datasets, performs any necessary preprocessing, creates appropriate database structures, and imports the data into the user’s choice of database management systems or text files. The automation of this process saves considerable time and substantially reduces the risk of errors resulting from repetitive procedures involving manipulation of the data by hand. This will enable ecologists to more easily use an array of existing data in their analyses, potentially leading to broader, more general, and more impactful research.

Currently, there are several other exciting ecoinformatics initiatives underway, which differ in aim and scope from the EcoData Retriever but can be used in combination with the Retriever to address the overarching problems of data access and publication. Most of these efforts focus on creating centralized repositories for data and metadata (e.g., Dryad, http://datadryad.org; The Knowledge Network for Biocomplexity, http://knb.ecoinformatics.org; DataONE, https://www.dataone.org), but do not provide tools for quickly installing the data in a well structured form for local use. This is the niche that is filled by the Retriever.

The broadest current informatics initiative in ecology is DataONE (https://www.dataone.org), which aims to create a distributed network for the publication of scientific data and metadata from a broad array of scientific disciplines, and eventually to facilitate the local installation of this data [14]. While this is a novel and useful solution to the problem of large-scale data access and storage, full implementation and widespread adoption of large cyberinfrastructure projects such as DataONE takes time and requires buy in from the broad array of data producers and providers. The EcoData Retriever provides a simple, user-oriented system for accessing currently available data exactly as it is currently published. It does not aim to create a new data repository but instead works with any existing online source of data. Because the EcoData Retriever downloads data files that are already available for download over the web, little to no additional coordination with data owners is necessary. Data producers can continue to use whatever repositories and data publication resources they choose to publish their data. After posting data to one of these repositories a simple text script can be added to the Retriever (see below for a description of these scripts) to make it straightforward for others to start using the data immediately. These scripts typically required no programming background allowing any data depositor to include their data in the Retriever. The EcoData Retriever offers a simple solution for quick data discovery and access.

## Description

The EcoData Retriever (http://ecodataretriever.org), is written in the Python programming language and is designed to be modular and easily extensible to address the varied data needs of researchers by allowing new datasets and new database management systems to be easily added. Binary packages are available for Windows and Ubuntu/Debian Linux, and the Retriever can also be built directly from the source code to work on any platform. The source code has been released under the MIT license (http://www.opensource.org/licenses/mit-license.php) and can be downloaded from GitHub (https://github.com/weecology/retriever) or from the project website. The Retriever currently provides support for MySQL, PostgreSQL, Microsoft Access and SQLite database management systems, as well as exports into comma-delimited text files. Once the Retriever places in the data in a database management systems it can be easily extracted in whatever form is necessary for a specific analysis using queries, and most programming languages, including R, Python, and Matlab, can directly query data from these database management systems. In addition, relational database management systems allow multiple datasets to be easily combined. Alternatively, the text files can be opened directly in common data analyses programs such as R or Microsoft Excel. The Retriever requires an active internet connection to download the data. It is not dependent on any commercial software packages.

Currently over 20 datasets are available via the EcoData Retriever (Table 1). This includes several major datasets that are only available through their own, unique, online source such as the Forest Inventory and Analysis Data (http://www.fia.fs.fed.us/), the Alwyn Gentry Forest Transect Data (www.mobot.org/MOBOT/research/gentry/transect.shtml), USDA plants taxonomy data (plants.usda.gov) and the North American Breeding Bird Survey (https://www.pwrc.usgs.gov/bbs/), as well as a number of datasets from Ecological Archives (http://esapubs.org/archive/default.htm). See Table 1 for a full list of datasets that can be currently be acquired using the EcoData Retriever,

**Table 1:** A sample of datasets available from the EcoData Retriever.

**Dataset Name**	**Size**	Download & Installation Time*
Capellini et al. 2010 [21]	1 file, 55.3 KB	1 second
Petraitis et al. 2008 [24]	2 files, 121 KB	1 second
Ernest et al. 2003 [6]	1 file, 149.6 KB	1 second
Smith et al. 2003 [26]	1 file, 372 KB	2 seconds
Lislevand et al. 2007 [4]	1 file, 824.5 KB	5 seconds
Jones et al. 2009 [7]	1 file, 2.2 MB	9 seconds
USDA Plant Taxonomy	1 file, 6.9 MB	16 seconds
McGlinn et al. 2010 [23]	6 files, 1.5 MB	16 seconds
Ramesh et al. 2010 [25]	4 files, 1.6 MB	18 seconds
North American Breeding Bird Survey [2]	66 files, 217.2 MB	18 seconds
Ernest et al. 2009 [9]	3 files, 2.1 MB	23 seconds
Woods 2009 [27]	6 files, 2.3 MB	25 seconds
Del Moral 2010 [10]	4 files, 485.6 KB	28 seconds
Zachmann et al. 2010 [28]	1 file, 10.1 MB	35 seconds
Adler et al. 2007 [19]	6 files, 10.1 MB	40 seconds
Alwyn H. Gentry Forest Transect Data	226 files, 9.4 MB	44 seconds
Barnes et al. 2008 [20]	1 file, 21.5 MB	1 minute, 13 seconds
Forest Inventory and Analysis [3]	329 files, 6.5 GB	43 minutes, 31 seconds

* Tested using MySQL on a machine with 4 GB RAM and 4 x 2.4GHz processor.

Includes time required to download and reformat data and import to MySQL

The Breeding Bird Survey data provides a good example of the benefits of using the Retriever. This massive, continental-scale, dataset provides over 50 years of relative abundance information for over 1,500 species and subspecies of birds at thousands of sites across North America, and is frequently used in ecological research [15,16]. The database consists of multiple tables; the main table contains over 5 million individual records. These records are not available online in a single file, but can be accessed from the USGS in individual compressed files grouped by either region or taxon. When grouped by region there are a total of over 70 files. While the core files are consistently formatted, supplemental tables required to work with the data are posted in a variety of locations and formats. Previously, even experienced users hoping to use the entire Breeding Bird Survey database for analysis could expect to spend roughly a full day navigating the USGS website, downloading the data files, combining them, checking for errors, and importing the data. Doing these tasks manually leads to a significant likelihood of mistakes including invalid data types, missed imports, and files that were imported twice. Tasks that the EcoData Retriever automates in the case of the Breeding Bird Survey include: downloading all data files, extracting data from region-specific raw data files into single tables, correcting typographic errors, and adding a Species table that links species AOU numbers used by the Breeding Bird Survey to species names. The EcoData Retriever can acquire, format, and validate the data in approximately five minutes.

Additionally, the Retriever can assist researchers by restructuring complex or poorly structured datasets. One dataset whose use is greatly eased by restructuring is the Alwyn H. Gentry forest transect dataset. The data is stored in over 200 Excel spreadsheets, each representing an individual study site, and compressed in a zip archive. Each spreadsheet contains counts of individuals found at a given site and all stems measured from that individual; each stem measurement is placed in a separate column, resulting in variable numbers of columns across rows, a format that is difficult to work with in both database and analysis software. There is no information on the site in the data files themselves, it is only present in the names of the files. The Retriever downloads the archive, extracts the files, and splits the data they contain into four tables: Sites, Species, Stems, and Counts, keeping track of which file each row of count data originated from in the Counts table and placing a single stem on each row in the Stems table. Each of these tables contains data from all sites combined so that large-scale analyses on the entire dataset can easily be performed.

More generally, the EcoData Retriever handles a number of common tasks that need to be undertaken when working with ecological data. These tasks include: 1) creating the underlying database structures, including automatically determining the data types; 2) downloading the data from disparate sources across the web; 3) transforming data into appropriately normalized forms for database management systems (e.g., converting cross-tabulated data into the standard one record per line format and splitting tables into proper sub-tables to avoid duplicated data); 4) converting heterogeneous null values (e.g., 999.0, -999, NaN) into standard null values; 5) combining multiple data files into single tables; and 6) placing all related tables in a single database or schema.

While none of these tasks is inherently difficult to perform, the time and energy required to determine the basic structure and data types for a new database, learn the quirks and syntax of different database management systems, write the table creation scripts, and manipulate the raw data into standard structures, can end up representing a substantial fraction of the time and energy that goes into the analysis of a single dataset. When this is scaled to projects that analyze numerous large datasets simultaneously [17] this effort can begin to represent an impediment to including more data in ecological analyses. Automating this process allows scientists to focus their time and energy on doing science rather than on acquiring and manipulating data and should lead to an increase in the amount of data that is used and the rate at which large-scale ecological analysis and synthesis can be performed.

### Usage

**Figure 1:**
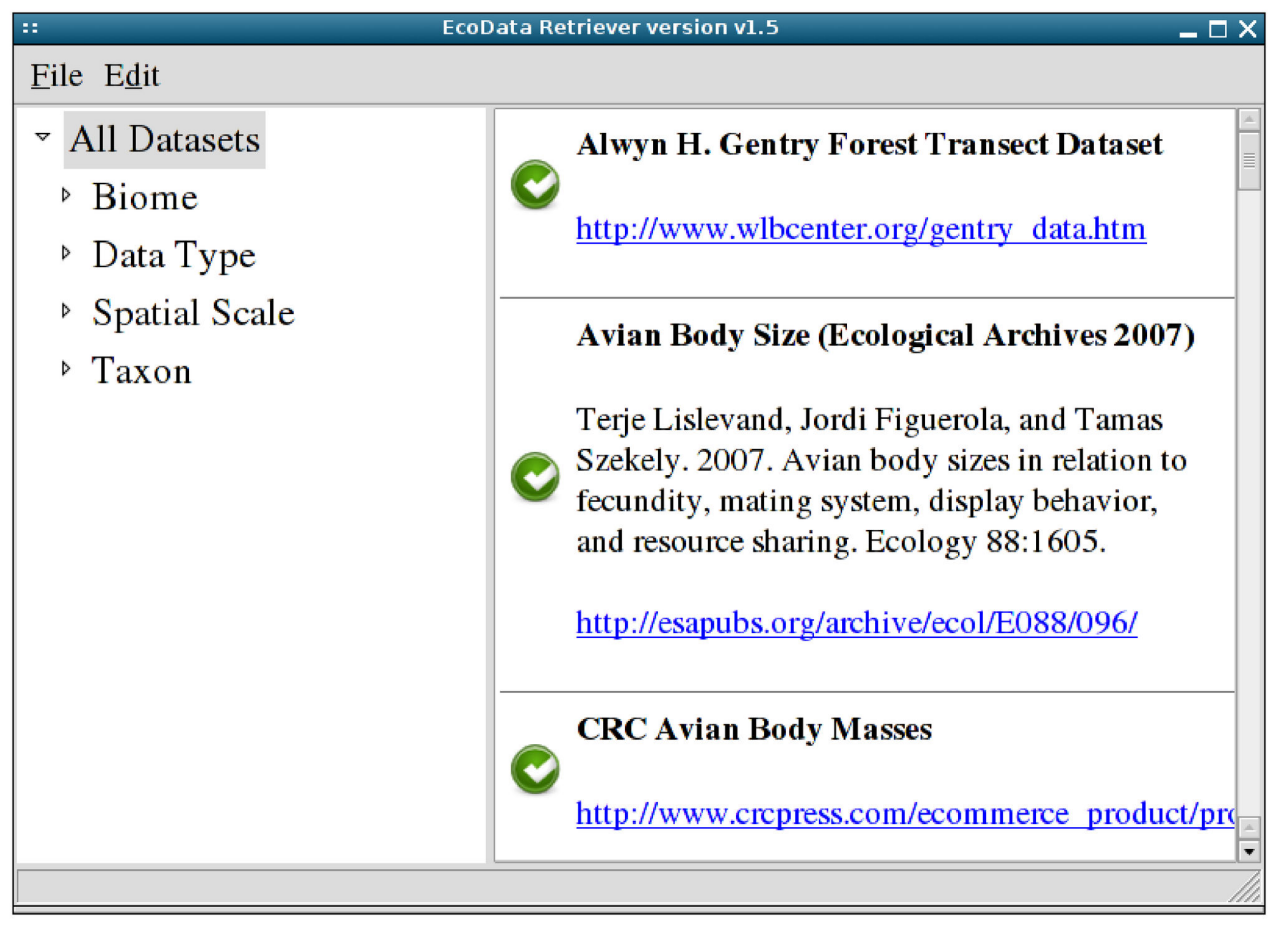
The EcoData Retriever dataset download interface. Each available dataset includes citation information as well as a link to more information from the dataset homepage.

The Retriever can either be run either using a graphical interface for easily selecting and downloading individual datasets, or from the command line to allow automated installation of datasets by other programs. The first time the Retriever is run the user is asked to choose a data management system (currently MySQL, PostgreSQL, Microsoft Access, SQLite, or comma-delimited text files), which will be used to store all of the data acquired by the EcoData Retriever. This setting can be changed if the user wants to store different datasets in different ways. After entering the information needed to connect to this data source, the main interface is displayed, providing a list of available datasets that can be filtered by selecting the category or subcategory of data that is of interest (Figure 1). Examples of such categories available for filtering data include taxon and spatial scale. Citation information and links to additional information about the dataset are also provided.

The icon to the left of each dataset displays the status of the dataset: a green check mark means that the data has been successfully acquired and is already present in the specified database; an open box signifies that the dataset is available for download; and a red X means that an error has occurred. Downloading and importing a dataset is as simple as clicking on the icon or double-clicking on the dataset. Behind the scenes, the Retriever will connect to the external data source, download the data files, perform any necessary restructuring for the dataset, and import the data. The user will be updated as these tasks are completed, and, when the data is available, the dataset icon will change to a green check mark. The data is then available in the selected location, andto be accessed with the user’s choice of data manipulation tools. For example, the text files can be imported directly into Excel or R, or the databases can be queried from inside of R using packages like RMySQL (http://cran.r-project.org/web/packages/RMySQL/index.html) or RPostgreSQL (http://cran.r-project.org/web/packages/RPostgreSQL/).

The Retriever also includes a command line interface (CLI) to allow it to be utilized in research workflows and pipelines. For example, the BBS data can be imported into an SQLite database named projectdata.sqlite by running retriever install BBS -e s -f projectdata.sqlite from a command prompt. More details on the CLI are available at the project website (http://ecodataretriever.org).

### Program design

**Figure 2:**
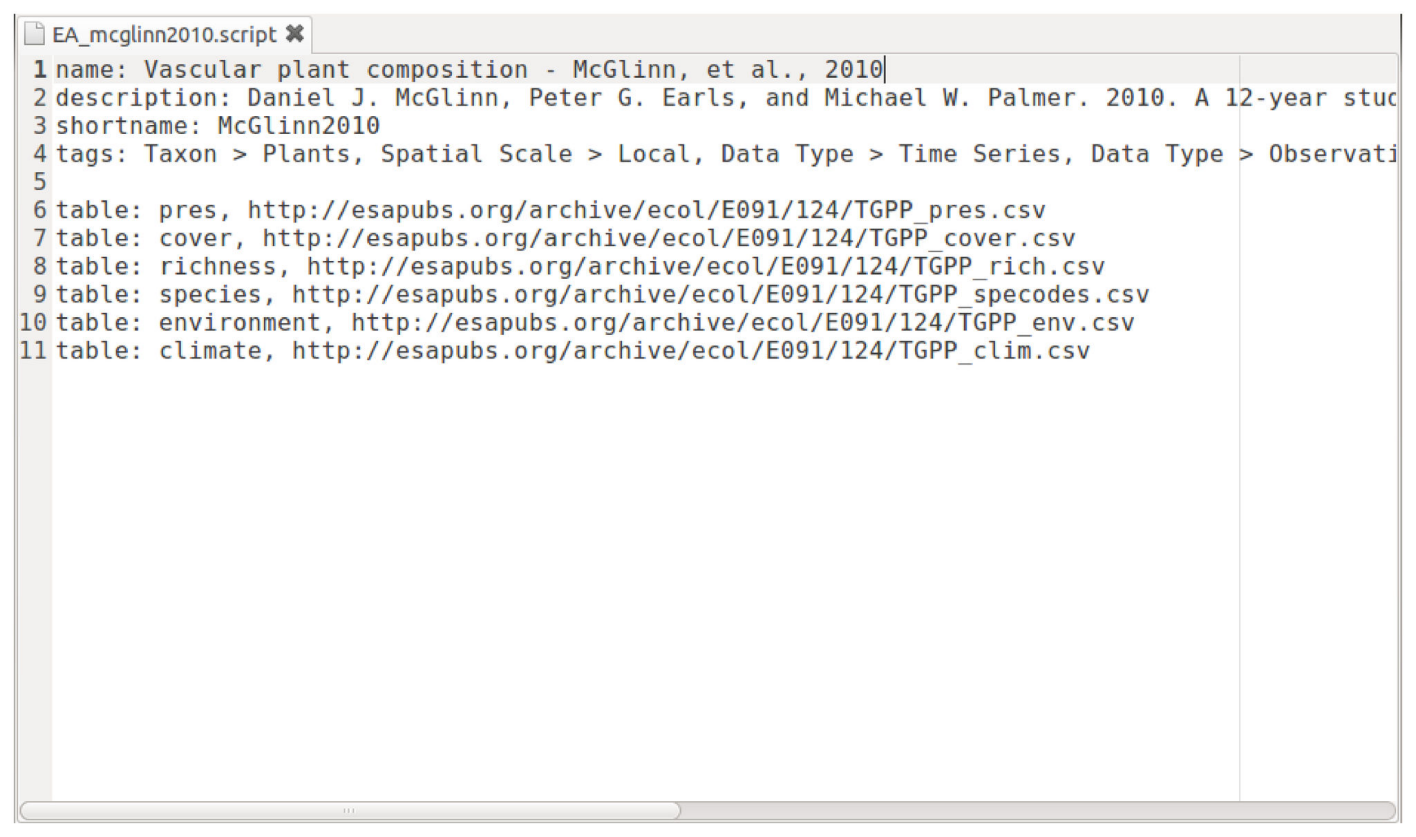
An EcoData Retriever dataset script file. An example of a simple EcoData Retriever dataset script file for a dataset containing six tables. For many text-based data formats, the EcoData Retriever will automatically infer column names and data types from the data file itself, so users need only to list the data file URLs and metadata such as name and citation.

The EcoData Retriever combines three components: 1) the main application, which manages all of the standard tasks related to downloading, preprocessing, and structuring the data; 2) a set of database management system engines that allow the Retriever to communicate with the different kinds of database software; and 3) scripts that store the information necessary to acquire and format individual datasets. The dataset information for most simple data files is stored in simple text files (see Figure 2 for an example of a text-based script), which allow the software to be quickly extended to include more datasets. These text files are also a convenient way to catalog metadata. This simple text format can import multiple tables (as long as each table is in a single file, standardize null values, set or change field names and data types, and restructure cross-tab data into standard database format. Scripts for datasets requiring more substantive manipulation or more complex data structures are written using the Python programming language, allowing any degree of complexity in the raw data to be handled effectively.

The extensible design of the system makes it easy to add both new datasets and new database management systems to the Retriever. When the program is first started, it automatically downloads the latest versions of all dataset scripts from the Retriever’s online repository, so that it is not necessary to update the program itself to add new datasets. New dataset scripts are added to the repository and the next time that users run the Retriever those scripts will appear. Individual users can even use the Retriever’s machinery to work with their own datasets privately by writing appropriate scripts and placing them in the Retriever’s 'scripts' directory. The objected oriented design of the database management system engines allows new database management systems to be added by requiring only that non-standard aspects of the database management system by overridden. This means that users who work with other database systems (e.g., Oracle, Microsoft SQL Server) can easily add this functionality to the Retriever, and that new database management systems that are not currently in common use can be added later to maintain the utility of the Retriever over time.

### Collaborative open-source development

One of the major advances in the development of free software has been the ability to include the user community in the development of the software itself. This allows much more to be accomplished than if development was driven by one or a few individuals. This is particularly relevant in scientific contexts where programming often represents only a small fraction of the responsibilities of those writing the code. Including the broader scientific community also allows the users to influence the direction of future development.

The objective of the EcoData Retriever is to enable easy access to useful ecological and environmental datasets. The benefit provided by the software increases as more datasets are included. As such, we have written the Retriever so that most datasets can be added using a script that requires only a few lines of text, and no programming experience, thus making it easy for users to add scripts for datasets that are not yet available. Users are encouraged to create their own scripts, using the existing scripts and documentation on the project website as guides, and contribute completed scripts for inclusion in the Retriever. This will allow the number of datasets supported to grow more rapidly than would be possible without community involvement. Developers familiar with Python are invited to contribute scripts for more complicated datasets and improvements to the software in general. The source code and documentation are available at http://ecodataretriever.org; the projected is hosted on GitHub (https://github.com/weecology/retriever), and pull requests are welcome.

## Discussion

One of the major impediments to the use of existing ecological data is the time and effort required to identify relevant datasets, understand their structure, acquire them, and manipulate them to make them usable for general analysis across datasets. The EcoData Retriever attempts to address this challenge by enabling users to quickly discover data of interest to them (through searches or filtering based on basic metadata about taxon, biome, spatial scale, and data type) and then quickly download and import those data in a format of their choice so that they can immediately begin analyzing the data.

In addition to making it easier to do research with existing data the Retriever also makes this research more reproducible. There is broad agreement that specific research results should be repeatable by those outside of the original group of researchers [18]. As computational research becomes increasingly prevalent, it has become more important that this research is replicatable, i.e., that it is possible to repeat the analysis and get back the same result. One of the challenges for fully replicatable research is recording the process of initial data acquisition and manipulation. Recording the version of the EcoData Retriever that is used to acquire the data, along with the date on which the data was downloaded, provides a complete characterization of the process used for data acquisition and initial manipulation. In addition, we are in the process of adding additional data provenance features to the Retriever that will automatically record this information in the metadata for the database or in comments in the text files.

The EcoData Retriever is free, open-source, software designed to automate the task of downloading, configuring, and installing publicly available ecological data. This substantially reduces the time, effort, and expertise required to start working with available data, reduces the risk of errors being introduced to the datasets due to the manual manipulation of the data, and improves the reproducibility of ecological research.
